# A global database on non-volant small mammal composition in natural and human-modified habitats

**DOI:** 10.1016/j.dib.2019.103842

**Published:** 2019-03-19

**Authors:** André Luís Luza, Catherine Helen Graham, Sandra Maria Hartz

**Affiliations:** a Programa de Pós-Graduação em Ecologia, Departamento de Ecologia, Prédio 43422, Instituto de Biociências, Universidade Federal do Rio Grande do Sul, Av. Bento Gonçalves 9500, Bairro Agronomia, CEP: 91501-970, Post-Office Box: 15007, Porto Alegre, Rio Grande do Sul, Brazil; b Swiss Federal Research Institute, WSL- Zürcherstrasse 111, CH-8903, Birmensdorf, Switzerland

**Keywords:** Didelphimorphia, Edge effect, Eulipotyphla, Fragmentation, Non-volant small mammal sampling, Rodentia

## Abstract

Non-volant small mammals, which include small-bodied representatives from several mammal orders, have been used as a model group to test the effects of habitat conversion and edge creation on biodiversity. Small mammals occupy a large variety of habitat types and vegetation strata, and have varied lifestyles and diets. They include species with slow-to fast-life history (the Etruscan shrew *Suncus etruscus* and European Hare *Lepus europaeus*, respectively) and with very specialized to very generalist habits and diets (the Atlantic bamboo rat *Kannabateomys amblyonyx* and house mouse *Mus musculus*, respectively). There are no databases with global coverage focusing on small mammal composition in natural and human-modified habitats and that include neglected natural habitats (e.g. grasslands and savannas). Here, peer-reviewed articles were searched in the primary literature to synthesize almost half century (1973–2017) of research on small mammal composition in natural forests, grasslands and their natural edges, and in five types of human-modified habitats (human-induced forest edges, human-induced grassland edges, crop fields, clear-cuts and tree plantations). The complete database includes information from 199 peer-reviewed articles. Presence data were obtained for 534 species (including 30 unidentified) in 551 sites distributed in 45 countries, 92 ecoregions, 10 biomes and six realms. Measurements of sampling effort and number of species records (number of individuals, captures) per habitat were also obtained, from which researchers can calculate a measure of abundance standardized by the sampling effort. The database will be useful for researchers interested in local-to broad-scale patterns of alpha- and beta-diversity in natural and human-modified habitats.

**Table:** Specifications table

Subject area	*Biology*
More specific subject area	*Ecology*
Type of data	*Table*
How data was acquired	*Bibliographic searches*
Data format	*Raw*
Experimental factors	*Data about non-volant small mammal species incidence and number of records in natural and human-modified habitats, and sampling design and effort per habitat were obtained from primary literature.*
Experimental features	*Bibliographic searches in primary literature*
Data source location	*Global coverage*
Data accessibility	*Data are available with this article.*
Related research article	*Suchomel, Josef; Purchart, Lubos; Cepelka, Ladislav. 2012. Structure and diversity of small-mammal communities of lowland forests in the rural central European landscape. European Journal of Forest Research, 131:1933–1941.*

**Table:** 

**Value of the data** • New and geographically replicated database with focus on small mammal composition in natural and human-modified habitats; • The data include the incidence and number of records of non-volant small mammal species measured with varied sampling methods and efforts; • Data clearly differentiate artificial pastures from natural grasslands and savannas; • The dataset allows comparisons of diversity between natural and human-modified habitats because it includes habitat-scale information on small mammal incidence, number of records and sampling procedures; • Researchers interested in the analysis of specific regions, habitat types, sampling methods and taxonomic groups can easily extract information from the database.

## 1 Data

Peer-reviewed articles were searched in the primary literature to synthesize almost half century (1973–2017) of research on small mammal diversity in natural forests, grasslands and their natural edges, and in five types of human-modified habitats (anthropogenic forest edges, anthropogenic grassland edges, crop fields, clear-cuts and tree plantations) (Table 1). Forest and grassland fragments, continuous remnants and advanced secondary-regeneration were considered natural habitats, because composition and richness differences among these habitats are minimal [1]. Managed forests were considered natural habitats when the authors provided enough information about logging regimes to judge that they were only minimally disturbed [2], [3]. Grasslands and savannas with native vegetation were considered natural habitats even if they were grazed by domesticated animals [4]. A human-induced edge was considered the boundary between natural and human-modified habitats. Species composition at edges was generally quantified with traps paralleling the sharp border between two habitats, mostly between forest and human-modified habitats [2], [3], [5]. Tree plantations considered here were those tree monocultures planted in grasslands and cleared forests. Finally, clear-cuts/young-secondary vegetation and crop fields were considered as two different types of open habitats (Table 1).

**Table 1:** Number of sampling units (habitats) per biogeographic realm.

	Afrotropic	Australasia	Indo-Malay	Nearctic	Neotropic	Palearctic	Total
Natural habitatsrowhead							
Forest	40	42	6	91	80	28	287
Grassland	15	0	0	59	25	17	116
Natural edge	1	0	0	3	0	0	4
Human-modified habitatsrowhead							
Forest edge	3	14	0	32	11	23	83
Grassland edge	1	3	0	5	7	5	21
Clear-cut	1	7	0	38	1	9	56
Crop field	10	6	0	26	17	12	71
Tree plantation	4	9	1	4	2	16	36
Total	75	81	7	258	143	110	674^a^

a The total number of sampling unities was calculated considering that each habitat within a site represents a different sample.

## 2 Experimental design, materials and methods

Bibliographic searches were used to obtain data on small mammal assemblage composition in natural and human-modified habitats. Peer-reviewed articles were searched in SCOPUS and ISI Web of Knowledge, according to indexed title, abstract, keywords and topics, using two search strings: 1) mammal* AND edge* AND forest*, and 2) mammal* AND edge* AND grassland* OR crop* OR field* (Table 2). The first set of key words returned few articles about native grasslands (Table 2). To better represent grasslands and their edges, additional searches were conducted (Table 2). In total, five of six bibliographic searches were considered because one of them returned no suitable article (Table 2). A total of 199 of the 1054 reviewed articles were included in the database because they provided enough information about the trapping techniques and effort used to sample non-carnivore, non-strictly forest species (e.g. Primates, Dermoptera) with an averaged body mass ≤ 5kg in natural and human-modified habitats.

**Table 2:** Number of research articles in each bibliographic search.

Database	Search order	Search topics (date of the last update)	Total number of research articles (% already included in the previous search)	Suitable peer-reviewed articles
Scopus	1°			
	mammal* AND edge* AND forest* (2017/26/07)		506	115
	2°			
	mammal* AND edge* AND grassland* OR crop* OR field* (2017/26/07)		364 (34%)	26
ISI Web of Science	3°			
	mammal* AND edge* AND forest* (2017/08/08)		609 (60%)	49
	4°			
	mammal* AND edge* AND grassland* OR crop* OR field* (2017/08/08)		3,082,493^a^ (−)	–
	5°			
	mammal* AND edge* AND grassland* OR cropfield* (2017/09/08)		124 (48%)	9
Scopus	6°			
	mammal* AND edge* AND grassland* OR cropfield* (2017/09/08)		63 (100%)	0
Total number of research articles			1054	199

a Not considered due to large number of articles from other sciences.

Most of data about site location, sampling details, species incidence and number of records were obtained from tables and text. Figures (except ordination diagrams) and axes values were interpreted to obtain sampling effort, species incidence and number of records when precise information was lacking in tables and text. Research articles in which the authors did not show information about the habitat occupied by at least one species were not included in our database; the not available (“NA”) entry was used for species lacking habitat information. To minimize the amount of missing data, authors were contacted to obtain information omitted in the published articles (see Acknowledgements for the list of authors). If no detail was provided by emailing the authors the not available entry was maintained. Also to minimize the amount of missing data, in one case (Hutchison and Rodgers [6]) data were took from a thesis because the published article was not found. In two cases (Lambert et al. [7], Lomolino & Smith [8]) data were obtained from other articles because these authors explicitly commented that the data were available in other publications. In two other cases [9], [10] data were extracted from the PREDICTS database [11] because data were better described in PREDICTS than in the original articles.

Most of research included in the database had the objective of evaluating small mammal habitat preferences between natural and human-modified habitats and comparing assemblage richness and composition between these habitats [2], [5]. Researches assessing the effect of habitat conversion and creation of edges on medium and large-sized mammals with information on the distribution of mammals weighing ≤5 kg [12] were included as well. Finally, researches assessing the effect of habitat conversion and creation of edges on prey availability [13], predation of nests [14], predation of saplings, fruits, seeds and invertebrates [15], small mammal populations (when presenting information of coexisting species) [9] and on the risk of diseases due to the incidence of mammalian vectors [16] were also included in the database. The composition of non-volant small mammal assemblages in natural and human-modified habitats was generally assessed using trapping grids or transects in homogeneous habitats (e.g. one grid or transect in the forest interior, one in the grassland interior, and one in the human modified-habitat [2]) or across habitats (e.g. grid or transect from forest interior until grassland interior [4], [5]). The database can be found in Appendix S1 and the complete list of references can be found in Appendix S2.

The main objective of the database was to provide information about the spatial distribution of non-volant small mammal species in natural and human-modified habitats. Thus, information about sampling effort, assemblage composition and species abundance obtained over many sampling occasions (e.g., over different seasons, years) was summed and summarized in a unique sampling occasion. By summarizing the temporal information, a species was considered present in a given habitat if it was recorded in at least one sampling occasion.

Defining the location of sites and habitats was not always trivial. Coordinates provided by the authors were generally imprecise (e.g., 51°5′N, 9°9′E) and in different unities (e.g., UTM, decimal degrees) (Table 3). The location of the coordinates indicated by the authors were checked and transformed into decimal degrees with as many decimal places as possible. In cases where the authors did not show the specific location of sites and local habitats, coordinates were searched in Google Earth based on any locality name provided by the authors. Coordinates of the region or site were used when coordinates of local habitats were lacking.

**Table 3:** Description table.

Descriptor	Type of descriptor	Levels/unit of descriptors	Characterization of the levels
SCOPUS_WEB_SEARCH	Categorical	Five levels:	Descriptor characterizing in which bibliographic search an article was found:
		Forest	SCOPUS searches:
		Open	Forest (keywords: mammal* AND edge* AND forest*);
		Forest/open	Open (keywords: mammal* and edge* and grassland* or crop* or field*);
		ForestWEB	Forest/open: peer-reviewed articles found in both “Forest” and “Open” searches.
		GrasslandWEB	WEB OF SCIENCE searches:
			ForestWEB (keywords: mammal* AND edge* AND forest*);
			GrasslandWEB (keywords: mammal* and edge* and grassland* or crop* or field*)
REFERENCE	Categorical	199 data sources (peer-reviewed research articles)	The citation of the articles included in the database. References in Appendix S2.
COUNTRY	Categorical	45 levels	The country where the research was conducted.
REGION	Categorical	186 levels	Information of the region where the research was conducted.
SITE	Categorical	409 levels	Sites (localities) sampled within a region.
			The research articles including only one unnamed site were named as *unique*, or those articles for which it was not possible to define different sites. We named the sites using some designation provided by the authors (e.g., name of treatments within an experiment).
ECO_CODE	Categorical	92 levels	A code for each ecoregion. The code is composed by the code of each realm (first two characters), the code of each biome (third and fourth characters) and the code of each ecoregion (fifth and sixth characters).
ECO_NAME	Categorical	92 levels	The complete name of each ecoregion
ECO_NUM	Numerical	41 codes	The code of each ecoregion
WWF_REALM	Categorical	Six levels	The acronym of each realm.
WWF_REALM2	Categorical	Six levels	The complete name of each realm
WWF_MHTNUM	Numerical	10 codes	The code of each biome
WWF_MHTNAM	Categorical	10 levels	The complete name of each biome
N_YEAR	Categorical	10 levels	The number of occasions and temporal replication of sampling. Two_seasons: sampling was conducted continuously during two or three seasons. Sampling did not cover one year;
			snapshot: one discrete and quick sampling, without temporal replication;
			one_year: continuous sampling lasted at most one year;
			many years: continuous sampling over many years (apparently without discrete periods of sampling);
			two_snapshots: more than one discrete snapshot was conducted in the same season;
			mon_snapshot_year: seasonal sampling where monthly discrete snapshots were conducted during more than one year;
			two_year_snapshot: discrete snapshots conducted in two or more years in similar seasons (i.e., at least one temporal replication);
			mon_snapshot: discrete snapshots in sequential months but total sampling did not cover one year;
			two_season_snapshot: discrete snapshots conducted in two or more seasons of the same year;
			NA: information not available.
SNAPSHOT	Binary	Two levels-	Description if sampling was temporally replicated (1) or not (one discrete snapshot, 0). NA: information not available.
SEASON_OF_TRAPPING	Categorical	23 levels	The season (s) in which the sampling occasion (s) occurred. Depending on the “N_YEAR” descriptor, “SEASON_OF_TRAPPING” describes either one season (we provide the name of the season) or many seasons (e.g., “spring_summer”, “many_year_seasons”). The name and number of the seasons depends on the region where the research was conducted (subtropical regions: autumn, winter, spring, summer; tropical regions: dry and wet seasons). NA: information not available.
ONE_SEASON	Binary	Two levels-	If sampling was conducted in one (1) or more seasons (0). NA: information not available.
EFFORT_PER_HABITAT	Numerical	Several types	Value expressing the total sampling effort per habitat. NA: the sampling effort could not be calculated using the information provided by the authors.
EFFORT_UNIT	Categorical	13 levels	Depending on the sampling method, sampling effort can be in trap-nights, camera-days, number of scats, number of owl pellets, kilometers monitored, number of hours or days travelled in transects. NA: the sampling effort unit could not be defined.
TRAP_TYPE	Categorical	65 levels	The name of the method used to record the species, including: box-like traps: sherman, wooded chmela, longworth, BTTm, triptrap, and elliott live-traps;
			snap-like traps: Victor snap, spring traps, Museum snap, fenn trap, mouse and rat snap traps;
			wire meshed traps: tomahawk, wire cage, mascot, ugglan, havahart;
			traces: methods to register tracks and bites (artificial eggs, hairtubes, sandplots, trackplates, snowtracks, tracking tunnels, transects, visual sighting, spotlight, sandplots);
			pitfall traps: buckets connected or not by drift-fences;
			scats: species presence in owl-pellets, dogscats, catpreys;
			cameratrap: camera-traps (pictures, videos);
			NA: method not available
BOX.LIKE	Binary	Two levels	Mammal sampling using sherman, wooded chmela, longworth, BTTm, triptrap, and Elliott live-traps (1) or not (0). NA: information not available.
SNAP.LIKE	Binary	Two levels	Mammal sampling using Victor snap-traps, spring snap-traps, Museum snap-traps, fenn snap-traps, mouse and rat snap-traps (1) or not (0). NA: information not available.
WIRE_MESHED	Binary	Two levels	Mammal sampling using tomahawk, wire cage, mascot, ugglan and havahart wire-traps (1) or not (0). NA: information not available.
TRACES_SIGHTS	Binary	Two levels	Mammal sampling using artificial eggs, hairtubes, sandplots, trackplates, snowtracks, tracking tunnels, transects, visual sights, spotlight, sandplots (1) or not (0). NA: information not available.
PITFALL	Binary	Two levels	Mammal sampling using plastic buckets connected or not by drift-fences (1) or not (0). NA: information not available
SCATS	Binary	Two levels	Mammal sampling by analyzing prey remains in owl-pellets, regurgitations or scats (1) or not (0). NA: information not available.
CAMERA	Binary	Two levels	Mammal sampling using camera-traps (1) or not (0). NA: information not available.
LAT_ORIGINAL	Numerical	Several types	Original latitude values provided by the authors. NA: information not available.
LONG_ORIGINAL	Numerical	Several types	Original longitude values provided by authors. NA: information not available.
LAT	Numerical	Decimal degrees	Latitude value in degrees after checking the location of sites
LONG	Numerical	Decimal degrees	Longitude value in degrees after checking the location of sites
SPECIES	Categorical	534 species (including 30 non-identified species)	Binomial name (or genus plus ‘sp.’ when the species was not identified). We used the nomenclature of Wilson & Reeder 2005 (also used by IUCN and Catalogue of Life).
ORDER	Categorical	13 levels	The mammalian orders
HABITAT	Categorical	Six levels	Habitat where species were registered edge – habitat edges
			forest – natural forests
			grassland – natural grasslands
			open – anthropogenic habitats with open vegetation structure
			tree_plantation – tree plantations
			NA – information not available
CLEAR_CUT	Binary	Two levels	If the HABITAT “open” is a clear-cut (1) or a crop field (e.g. soybean field, hayfield, artificial pasture) (0).
FOREST_EDGE	Binary	Two levels	If the HABITAT “edge” is the boundary of a forest (1) or grassland (0)
GRASSLAND_FOREST_EDGE	Binary	Two levels	If the HABITAT “edge” is a natural edge between natural habitats (1) or an human-induced edge (0)
FRAGMENTED	Binary	Two levels	Sampling was conducted in fragments of forests or grasslands (1) or it was conducted in continuous vegetation patches (e.g., conservation unities) (0). NA: information not available.
NUMBER_OF_RECORDS	Numerical	Several types	Number of records of a given species. NA: information not available.
PRESENCE	Binary	Two levels	Species presence (1). NA: information not available.
NUMBER_OF_RECORDS_UNIT	Categorical	11 levels	The unit of the number of records.
OBSERVATIONS	Categorical	–	Include information about how we calculated the sampling effort and number of records for some studies.

The total number of records and the sampling effort used to register the species are essential descriptors of data, because they can be used by researchers interested in calculating a measurement of species abundance that explicitly considers sampling effort. To enable this calculation, the total sampling effort and its unit were obtained from most of the reviewed articles. The sampling effort was registered in the same unit (trap-nights, camera-days) as showed in the research article. However, the number of records was not always directly related to the sampling effort. In many cases, the authors presented per-habitat number of individuals or captures but showed the total sampling effort instead of per-habitat sampling effort. It was also common to find articles where authors showed values of total number of individuals, captures, and captures per-unit-effort (CPUE) without mentioning if these values were calculated using the total sampling effort or per-habitat sampling effort. In these cases, the total sampling effort was divided by the number of sampled habitats assuming that the same sampling effort was used in different habitats. We divided sampling effort by the number of habitats to obtain values of sampling effort at habitat scale.

Sampling effort unit varied across articles due to the use of different sampling methods (e.g., trap-nights for live-traps, camera-days for camera traps, kilometers monitored for transects). As a consequence, the unit of the number of records was dependent on the sampling method used by the authors. The number of records per species was generally showed as the total number of individuals, number of photographs, averaged abundance (e.g., averaged at habitat scale, average abundance from occupancy models), site occupancy probability from occupancy models and as indexes such as captures per-unit-effort (CPUE), relative abundance, frequency of occurrence and number of individuals per hectare. To be included in the database with similar units, values of the number of records of a species in a given habitat was generally calculated by transforming captures per-unit-effort into the original scale (CPUE x total sampling effort/sampling effort unit [e.g., 100 trap-nights]) and by multiplying the number of individuals, frequency of occurrence and number of individuals per hectare by the sampling effort, number of sites and area (assuming that the number of records was similar across sites and areas). These calculations allowed to obtain a measure of total number of records per habitat in the same scale for all species. In some cases, the number of records was considered as not available because the authors showed the summed number of records per site instead of per habitat, which makes inaccessible information at habitat scale; this was the main reason for sending email to the authors. The unit of the number of records was defined based on whether individuals could be differentiated. When individuals could be differentiated, as in mark-recapture studies, the entry of the number of records was “number_of_individuals”; otherwise, the entry was, for example, “number_of_captures” and “number_of_detections” (see Table 3).

**Fig. 1:**
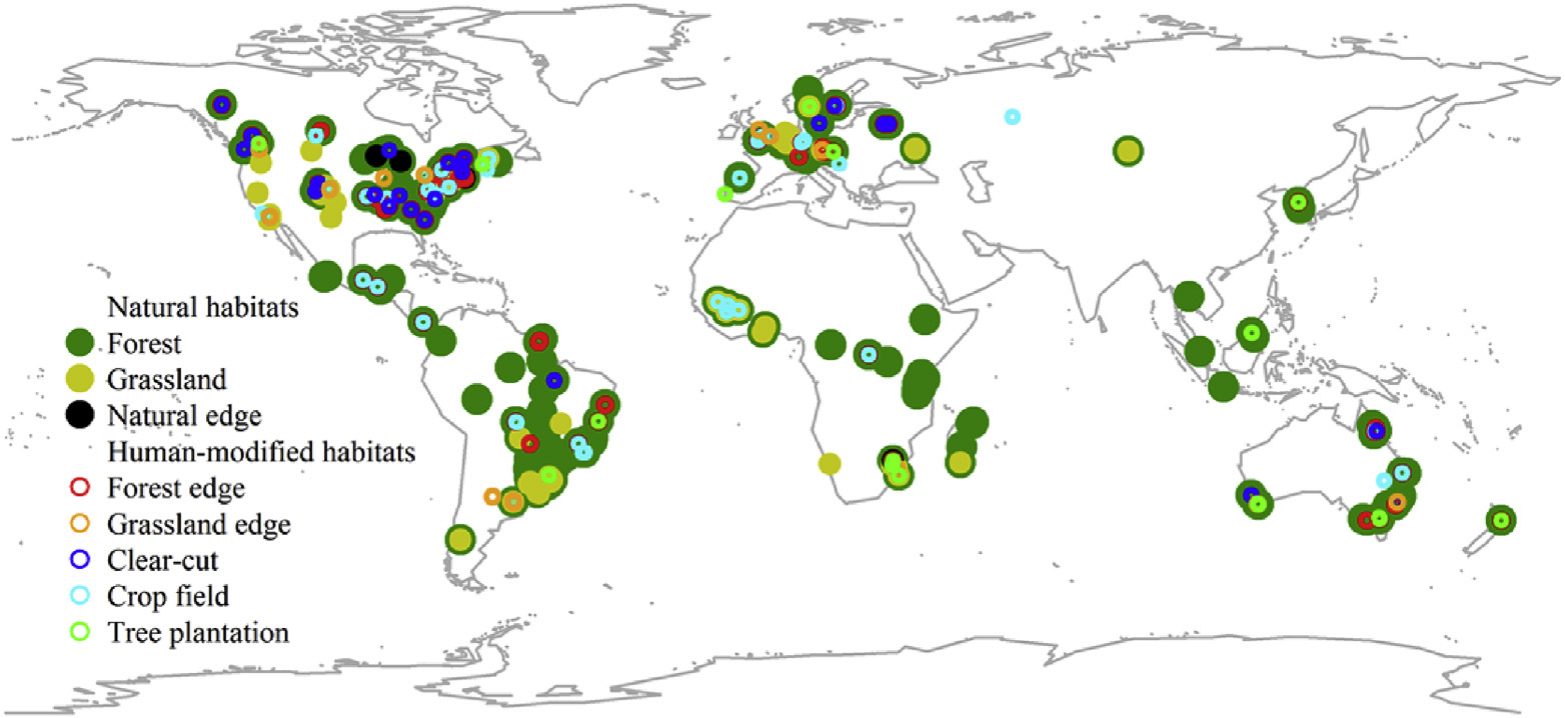
Global distribution of the sampled habitats.

The database has a global coverage, although more studies from the Neotropics, Nearctic and Palearctic (38%, 21% and 16%, respectively) were included in the database in comparison to Australasia, Afrotropics and Indo-Malay realms (12%, 11% and 1%, respectively) (Fig. 1, Table 1). It is interesting to note the lack of studies in the Indo-Malay realm and in eastern Palearctic (Fig. 1). The low number of studies in these regions probably occurs because the research is generally published in local journals with country-specific languages and is not indexed in platforms such as SCOPUS and ISI Web of Knowledge.

**Fig. 2:**
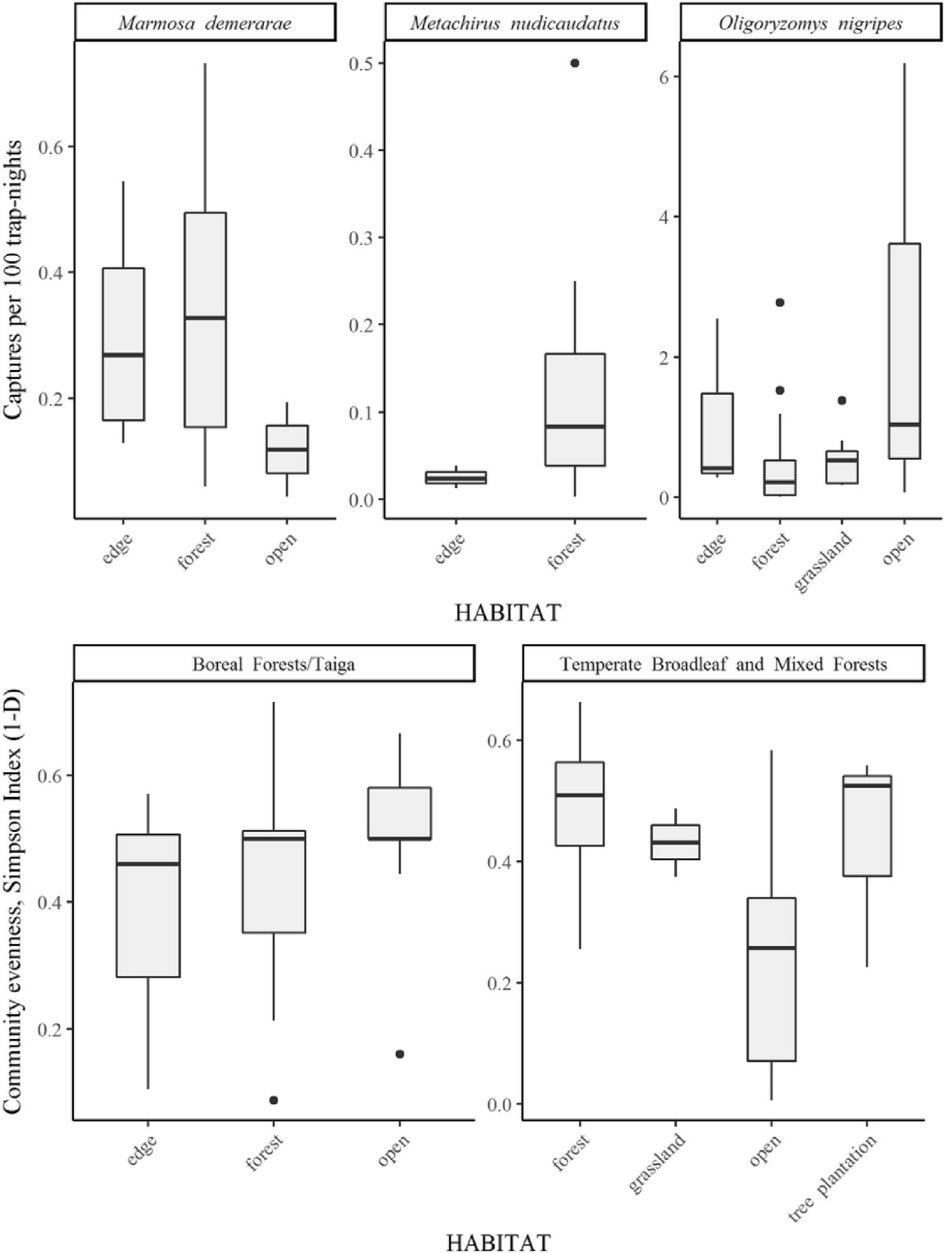
Per-habitat differences in the captures per 100 trap-nights of three Neotropical small mammal species (superior) and in the evenness of non-volant small mammal communities in two Nearctic biomes (inferior).

Researchers interested in the analysis of specific regions, habitat types, sampling techniques and taxonomic groups can easily extract information from the database. Here, the number of records and sampling effort were measured at local-habitat scale. Thus, researchers can filter species records according to a specific trapping method and habitat and then standardize the number of species records by the sampling effort, using indexes such as the captures per-unit-effort (number of records x total sampling effort/sampling effort unit [e.g., 100 trap-nights]). A brief analysis using the CPUE index with data obtained through live-traps and pitfalls showed that the most abundant species in natural forests were *Rattus fuscipes*, *Potorous tridactylus*, *Myodes gapperi*, *Peromyscus leucopus* and *Rattus leucopus* (2,263, 1,090, 1,066, 478 and 389 captures per 100 trap-nights, respectively), while in natural grasslands were *Microtus pennsylvanicus*, *Sorex cinereus*, *Mastomys natalensis*, *Peromyscus leucopus* and *Mus minutoides* (1,463, 1,162, 774, 337 and 300 captures per 100 trap-nights, respectively). Furthermore, researchers can use the data to compare the captures per-unit-effort of a particular species in different habitats, or compare the evenness of non-volant small mammal communities from different habitats (Fig. 2). The database will be useful for researchers interested in disentangling the processes governing the local-to broad-scale patterns of alpha- and beta-diversity in non-volant small mammal communities from natural and human-modified habitats.
